# Thermal Modelling Analysis of Spiral Wound Supercapacitor under Constant-Current Cycling

**DOI:** 10.1371/journal.pone.0138672

**Published:** 2015-10-07

**Authors:** Kai Wang, Liwei Li, Huaixian Yin, Tiezhu Zhang, Wubo Wan

**Affiliations:** 1 School of Automation Engineering, Dynamic integration and energy storage systems engineering technology research center, Qingdao University, Qingdao, Shandong Province, China; 2 College of Biological and Environmental Engineering, Zhejiang University of Technology, Hangzhou, Zhejiang Province, China; University of California Berkeley, UNITED STATES

## Abstract

A three-dimensional modelling approach is used to study the effects of operating and ambient conditions on the thermal behaviour of the spiral wound supercapacitor. The transient temperature distribution during cycling is obtained by using the finite element method with an implicit predictor-multicorrector algorithm. At the constant current of 2A, the results show that the maximum temperature appears in core area. After 5 cycles, the maximum temperature is 34.5°C, while in steady state, it’s up to 42.5°C. This paper further studies the relationship between the maximum temperature and charge-discharge current. The maximum temperature will be more than 60°C after 5 cycles at the current of 4A, and cooling measurements should be taken at that time. It can provide thoughts on inner temperature field distribution and structure design of the spiral wound supercapacitor in working process.

## Introduction

Oil depletion, growing mobility demand, and increasingly stringent regulations on pollutant emissions and carbon footprint are expediting a paradigm shift towards a sustainable and efficient transportation [1–5]. Supercapacitors, also referred to as Ultracapacitors (UCs) or electrochemical double-layer capacitors are energy storage devices between traditional electrostatic capacitors and batteries [6–11]. They have attracted worldwide attention because of outstanding advantages. Nowadays, supercapacitors have been widely used in hybrid cars, aviation aircrafts, and electronic communication, indicating broad application prospects [11–18].

Temperature, as one of the most important working parameters, has a great effect on stability of supercapacitors [19–22]. Temperature has a huge influence on supercapacitor cells and modules ageing. Because supercapacitors are high power electronic devices, heat will be generated and accumulated in inner region in rapid power absorption and release process, which can result in obvious temperature rise and even heat damage. Therefore, the study on the thermal stability of supercapacitors is very important. However, most of the research on thermal behaviors focuses on lithium ion batteries and Ni-H batteries, and the analysis on thermal behaviors of supercapacitors is relatively less [23–28]. Dae Hun Lee [19] proposed a three-dimensional symmetric thermal model of stackable supercapacitors based on the heating rate measured with calorimeter in charge-discharge process. He further studied the variation between inner temperature and ambient temperature according to the model. Hamid Gallous [20] studied the thermal behavior of spiral wound supercapacitors at small-medium power, and used built-in thermocouple to measure inner temperature rise. The results showed that the temperature rise varied with different charge-discharge currents, and it was even more than 60°C. Monzer Al Sakka [21] presents thermal modeling and heat management of supercapacitor modules for vehicle applications. The thermal model developed is based on thermal-electric analogy and allows the determination of supercapacitor temperature. Relying on this model, heat management in supercapacitor modules was studied for vehicle applications. Thus, the modules were submitted to real life driving cycles and the evolution of temperatures of supercapacitors was estimated according to electrical demands.

However, the inner temperature field distribution theory was not perfect, and he did not give out the specific temperature distribution of spiral wound supercapacitors in charge-discharge process [22–24].

Based on the above thoughts of thermal analysis on supercapacitors, this paper adopted the combination of finite element analysis and experiments to investigate the distribution of temperature change and inner temperature field at constant current to make supplements for former research and provide theoretical basis for the structure design.

The remainder of the paper is organized as follows: Section 2 describes Finite element modelling of supercapacitor. Section 3 introduces Thermal analysis theory of supercapacitor. Section 4 discusses the comparison results followed by the conclusions.

## Finite Element Modelling of Supercapacitor

**Fig 1:**
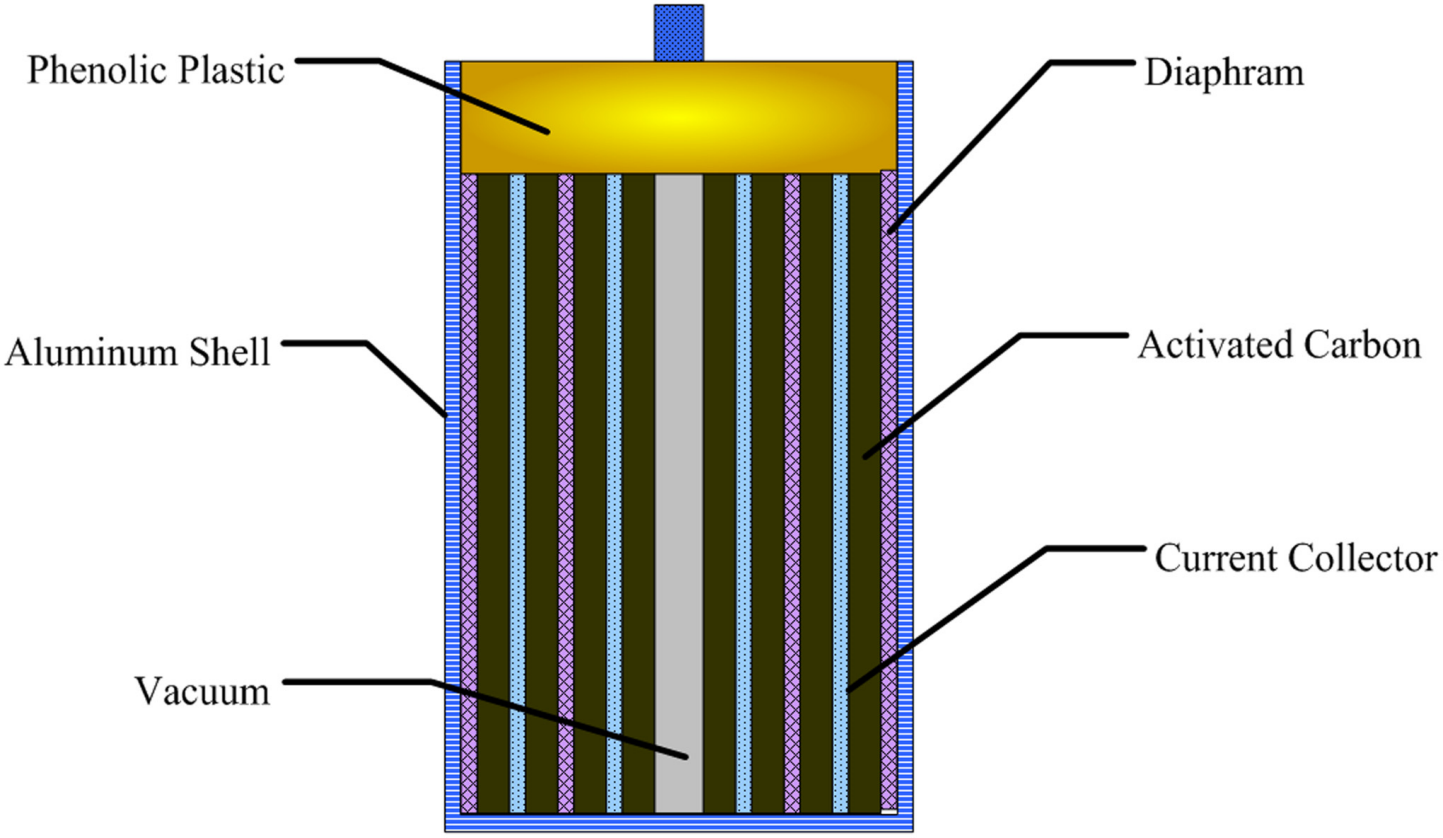
Structure schematic of supercapacitor.

In this paper, we assume the research object is the small spiral wound supercapacitor made by a domestic manufacturer, which is made up of aluminum shell, phenolic cover and inner part. Its dimension is 21mm×44mm (Diameter×Length, the height of lead wire is excluded). The structure is shown in Fig 1. The Supercapacitor is a certain domestic production of cylindrical winding type of SP-2R5 (KaiMei Power Limit Co.). The internal resistance was tested on the CHI608A electrochemical workstation (Shanghai Chenhua Limit Co.). The inner part is made up of activated carbon electrode, aluminum current collector and polypropylene diaphragm, with organic electrolyte system. The charge storage mechanism is electrical double layer capacitor storage principle [25–28], and the internal resistance is about 30mΩ. The material’s physical parameters (Table 1) are different.

**Table 1:** The physical characteristics of main material.

Material	Density kg·m^-3^	Specific Heat /J·kg^-1^·K^-1^	Thermal Conductivity/W·m^-1^·K^-1^		
			x	y	z
Electrode (Carbon)	1347.33	1437.4	1.04	1.04	237
PP	1008.98	1978.16	0.3344	0.3344	0.3344
Air	1.225	1006.43	0.03	0.03	0.03
Phenolic Plastic	1700	1700	0.5	0.5	0.5
Al Shell	2770	875	170	170	170

**Fig 2:**
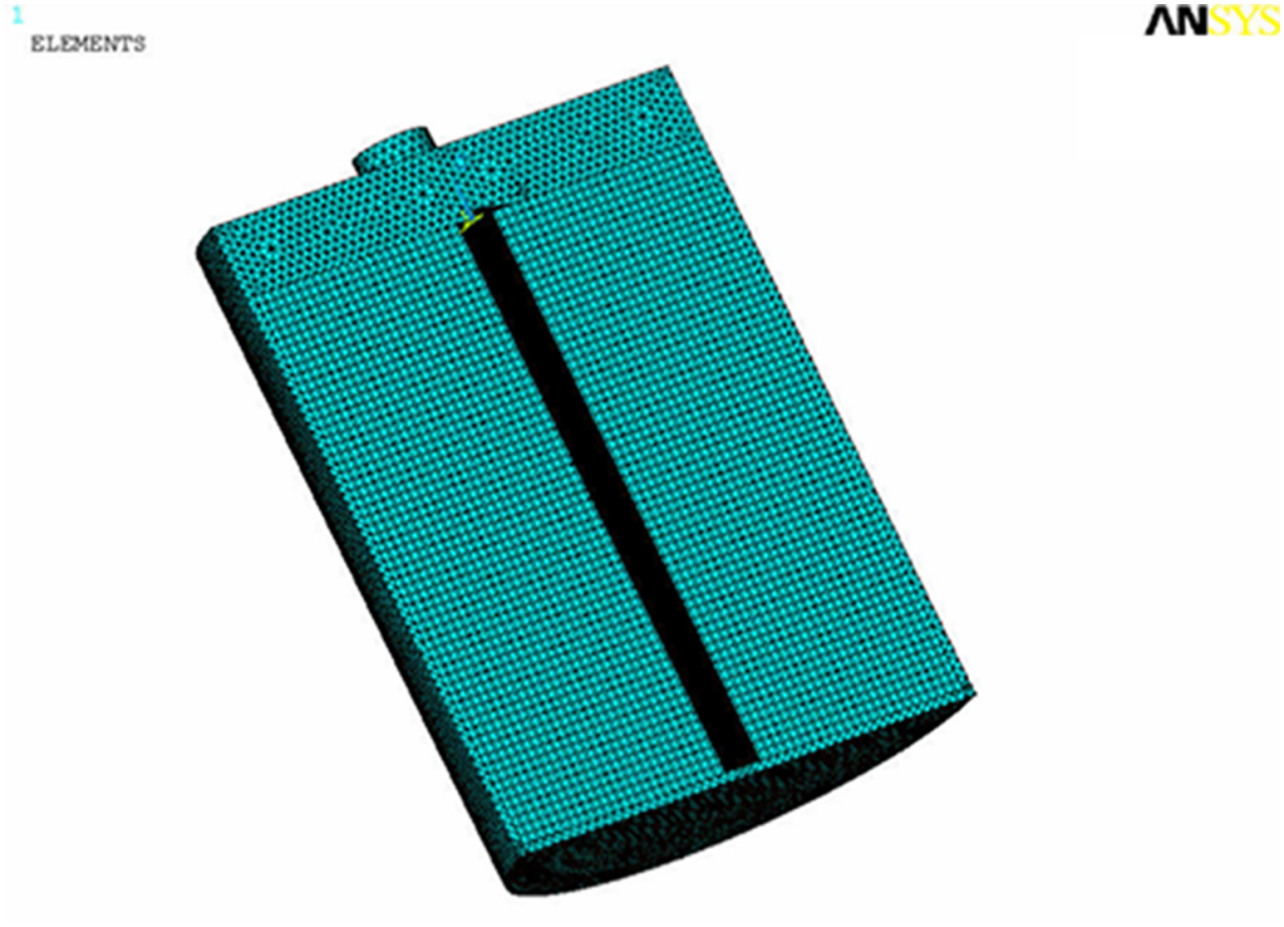
Finite element model of supercapacitor.

ANSYS is used to mesh the entity model (the result is shown in Fig 2). Hexahedron mesh is used in core and the more delicate tetrahedral mesh is adopted in shell, because shell is in the border region and involved in heat convection and radiation [18–20]. The whole finite element model consists of 408,679 units and 276,582 nodes.

## Thermal Analysis Theory of Supercapacitor

### Thermal analysis basic assumption

In the working process of supercapacitors, there are three ways of heat transfer, including heat conduction, convection and radiation. To simplify the analysis, these following hypotheses for the model have been raised [19–25]:

1. The simulation object is the electrical double layer supercapacitor and the charge storage principle is electrical double layer energy storage mechanism. Therefore the main form of inner heat is Joule heat generated by internal resistance.
2. In spite of the porous nature of activated carbon, the uneven distribution of electrolyte can result in uneven heat generation in inner region. However, at a holistic level, the heat is uniformly generated in inner region in charge-discharge process.
3. Ignoring electrolyte flowing in core, heat conduction can be seen as the only way for heat transfer there. On the contrary, heat radiation and convection are the two main ways on the surface of supercapacitor, because the effect of heat conduction is too weak to be considered.
4. According to characteristics of spiral wound supercapacitors and physical properties of different parts of the material, the radial thermal conductivity of carbon electrode could be approximately equivalent to the thermal conductivity of activated carbon, and the axial thermal conductivity be approximately equivalent to the thermal conductivity of aluminum current collector.

### Temperature distribution control equation

The transient temperature distribution of supercapacitor in working process can be described by the following equation [19]:

$$\nabla^{2}T+\frac{P}{\lambda }=\frac{\rho C_{\text{P}}}{\lambda }\frac{\partial T}{\partial t}$$ (1)

In this equation, ∇ is Laplace operator; *ρ* is density; *C*_P_ is specific heat capacity; *λ* is thermal conductivity; *P* is local volume density.

The research object in this paper is a cylindrical supercapacitor. For ease of description, the equation is changed into a three-dimensional cylindrical coordinates form:

$$\rho C_{\text{p}}\frac{\partial T}{\partial t}=\frac{\lambda_{\text{r}}}{r}\frac{\partial }{\partial r}\left(r\frac{\partial T}{\partial r}\right)+\frac{\lambda_{\theta }}{r^{2}}\frac{\partial^{2}T}{\partial \theta^{2}}+\lambda_{\text{z}}\frac{\partial^{2}T}{\partial z^{2}}+P$$ (2)

In this equation, *θ* is angular coordinate; *r* is radial coordinate; *z* is axial coordinate with conditions that 0°≤*θ*≤360°, *r*_i_≤*r*≤*r*_o_, 0≤*z*≤*L* and 0<*t*≤*t*_f_. *r*_i_ and *r*_*o*_ are the inside diameter and outside diameter of supercapacitor, respectively. *t*_f_ is local steady state temperature.

In working process, the thermal state is symmetrical in three-dimensional cylindrical coordinates, indicating that it has nothing to do with *θ*. Therefore, the formula can be simplified as follow:

$$\rho C_{\text{P}}\frac{\partial T}{\partial t}=\lambda_{\text{r}}\frac{\partial^{2}T}{\partial r^{2}}+\frac{\lambda_{\text{r}}}{r}\frac{\partial T}{\partial r}+\lambda_{\text{z}}\frac{\partial^{2}T}{\partial z^{2}}+P$$ (3)

### Definite conditions

To solve the temperature distribution control equation, corresponding definite conditions should be established. For transient thermal analysis, definite conditions have two aspects, namely giving out the initial conditions of initial temperature distribution and the boundary conditions of heat transfer. It can be respectively described as follow:

1. At initial time (*t* = 0), the temperatures of inner region and surface are uniformly distributed, which are the room temperature (25°C).
  $$T(r,z,0)=T_{\text{0}}$$ (4)
  In this equation, *r*_i_≤*r*≤*r*_*o*_, and 0≤*z*≤*L*.
2. On the innermost vacuum surface of supercapacitor core, it can be supposed that the surface is adiabatic and heat flux is zero because of the extremely low thermal conductivity. It can be obtained based on the Fourier law of heat conduction:
  $$\lambda_{\text{r}}\frac{\partial T}{\partial r}(0,z,t)=0$$ (5)
  In the equations, 0<*t*≤*t*_f_, 0≤*z*≤*L*.
3. On the outside surface of supercapacitor aluminum shell, methods of heat transfer are mainly heat convection which is conducted between air and the surface, and heat radiation with surroundings.

1) For heat convection, heat exchanging rate depends on convective heat transfer coefficient and the difference between surface temperature and ambient air temperature. It can be obtained based on the Newton's law of cooling:

$$q_{\text{conv}}=h_{\text{conv}}(T-T_{\infty })$$ (6)

In this equation, *T* is surface temperature; *T*_∞_ is ambient air temperature; *h*_conv_ is the convective heat transfer coefficient; *q*_conv_ is the heat flow rate per unit area of the convection heat transfer surface.

*h*_conv_ is affected by a lot of thermal physical parameters. The dimensionless Nusselt number *N*_u_ can be taken to quantitatively calculate it:

$$N_{\text{u}}=\frac{h_{\text{conv}}D}{\lambda_{\text{air}}}$$ (7)

In the equation, *D* is outside diameter of supercapacitor; *λ*_air_ is thermal conductivity of ambient air. The dimensionless Nusselt number *N*_u_ can be expressed as a function of another dimensionless Renault number *R*_e_:

$$N_{\text{u}}=CR_{\text{e}}{}^{n}$$ (8)

In this equation, dimensionless constant *C* and index *n* are measured through experiments.

The dimensionless Renault number *R*_e_ can be further transformed into the product of two dimensionless Planck number *P*_r_ and Grashof number *G*_r_:

$$R_{\text{e}}=P_{\text{r}}\times G_{\text{r}}$$ (9)

$$P_{\text{r}}=\frac{\eta_{\text{air}}C_{\text{p,air}}}{\lambda_{\text{air}}}$$ (10)

$$G_{\text{r}}=\frac{g\alpha (T-T_{\infty })D^{3}}{\upsilon^{2}{}_{\text{air}}}$$ (11)

In this equation, *η*_air_ is air kinetic viscosity; *C*_p,air_ is specific heat capacity of air; *g* is acceleration of gravity; *α* is volume expansion coefficient; *υ*_air_ is movement of air viscosity.

The heat convection of the outside surface of supercapacitor conforms to the natural heat convection principle of cylinder in a large space. According to the range of *R*_e_, *N*_u_ can be expressed as follow:

$$N_{\text{u}}=0.53R{\text{e}}^{1/4}\;(10^{3}\leq R\text{e}\leq 10^{9})$$ (12)

$$N_{\text{u}}=0.10R{\text{e}}^{1/3}\;(10^{9}\leq R\text{e}\leq 10^{13})$$ (13)

2) For heat radiation, radiance depends on *T* ^4^ (*T* is the thermodynamic temperature of supercapacitor surface) and surface emissivity. It can be specifically described based on the Stefan-Boltzmann law:

$$q_{\text{rad}}=\varepsilon \sigma (T^{4}-T^{4}{}_{\infty })$$ (14)

In the equation, *ε* is surface emission rate of aluminum shell; *σ* is the Stefan-Boltzmann constant (*σ* = 5.67×10^-8^Wm^-2^K^-4^).

It can be changed as follow:

$$q_{\text{rad}}=h_{\text{rad}}(T-T_{\infty })$$ (15)

In the equation, *h*_rad_ is the radiation heat transfer coefficient, and it can be defined as follow:

$$h_{\text{rad}}=\varepsilon \sigma (T+T_{\infty }\text{)(}T^{2}+T^{2}{}_{\infty })$$ (16)

Therefore, the total heat transfer coefficient of supercapacitor surface is:

$$h_{\text{c}}=h_{\text{conv}}+h_{\text{rad}}$$ (17)

Finally, the total heat flux is:

$$q=h_{\text{c}}(T-T_{\infty })$$ (18)

## Results and Discussions

**Fig 3:**
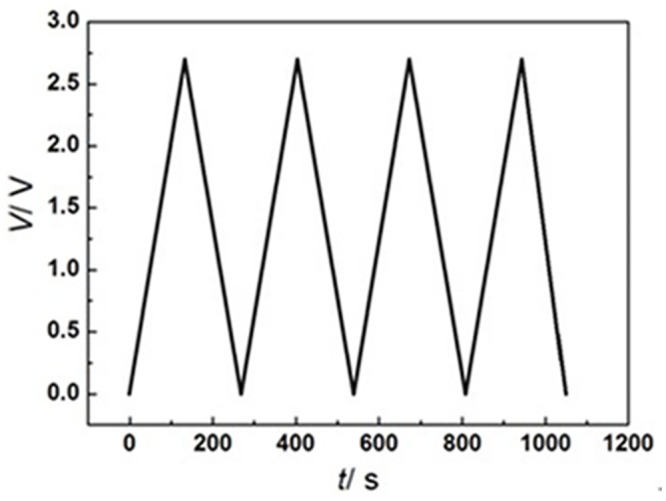
Curve of voltage profile changing with time.

At the room temperature (25°C), the model supercapacitor is charged and discharged at the constant current of 2A, and the variation between voltage and time is shown in Fig 3. In the temperature range of simulation, the internal resistance can be seen as a constant. As a result, its internal heating power is *Q* = *I* ^2^ *R* = 0.12W; the core volume is *V* = 1.97cm^3^; the heat generation rate per unit volume is *p* = 6.093×10^4^W/m^3^. The temperature is measured by the K type adhesive type thermocouple measurements (OMEGA).

Based on the thermal analysis theory and thermal physical parameters of the supercapacitor, its complex heat transfer coefficients (Table 2) are changing in the temperature range of simulation.

**Table 2:** Complex heat transfer coefficient.

*T*	28°C	31°C	34°C	37°C	40°C	43°C
*h*_conv_	4.356	5.175	5.727	6.151	6.496	6.802
*h*_rad_	1.523	1.546	1.571	1.594	1.618	1.642
*h*_c_	5.879	6.721	7.298	7.745	8.114	8.444

**Fig 4:**
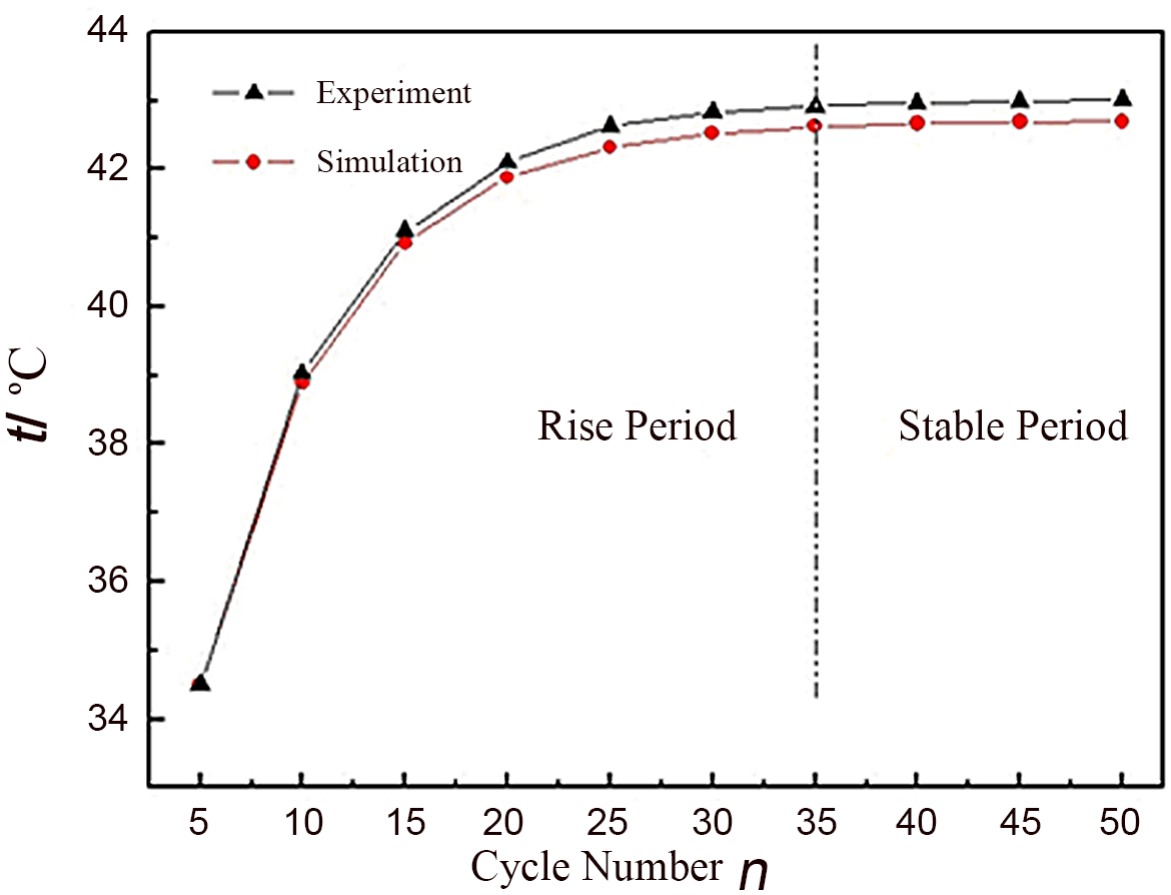
Maximum temperature in core area with the change of cycle times.

We have studied the variation between the maximum temperature and cycle number after 50 cycles. K-type thermocouple is adopted to measure the temperature, and the curves of experiments and simulation are shown in Fig 4 (see S1 File for details). The two curves can be roughly divided into two parts: rise period and stable period. They rise quickly at initial stage and tend stable after 35 cycles at 42.9°C and 42.5°C, respectively. Though there are some tiny deviations between experiment and simulation curves, they are consistent with each other generally. In addition, there is only one heat source in the simulation, which is the Joule heat produced by the internal resistance. As a result, the simulation is reliable.

**Fig 5:**
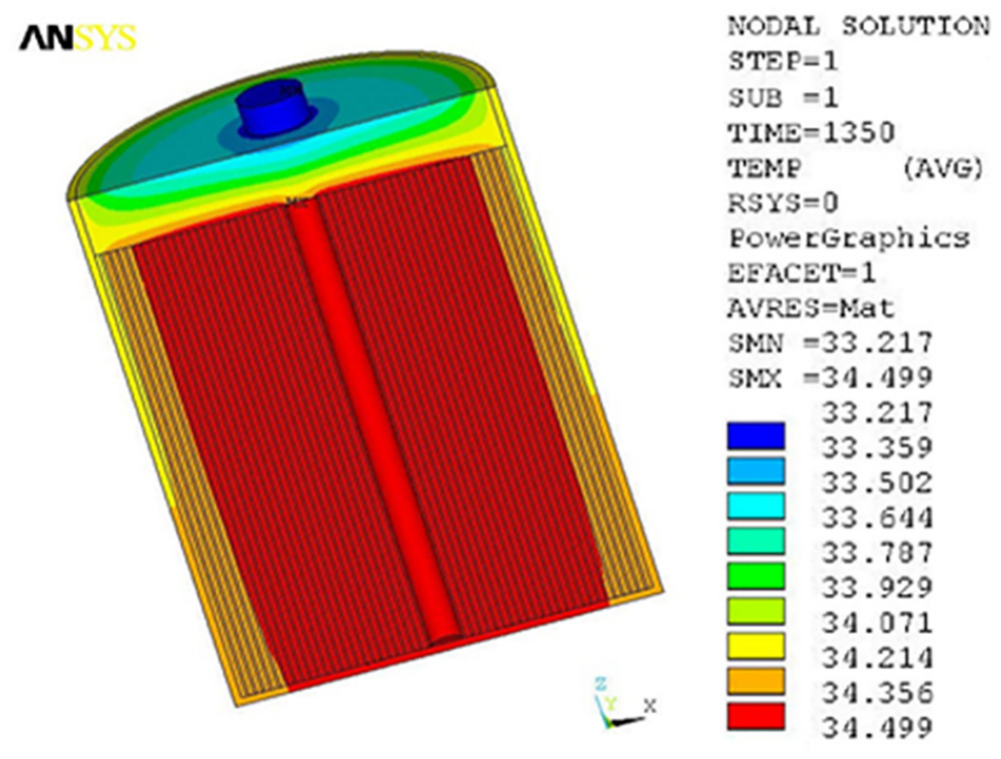
Temperature distribution of 5 cycles.

**Fig 6:**
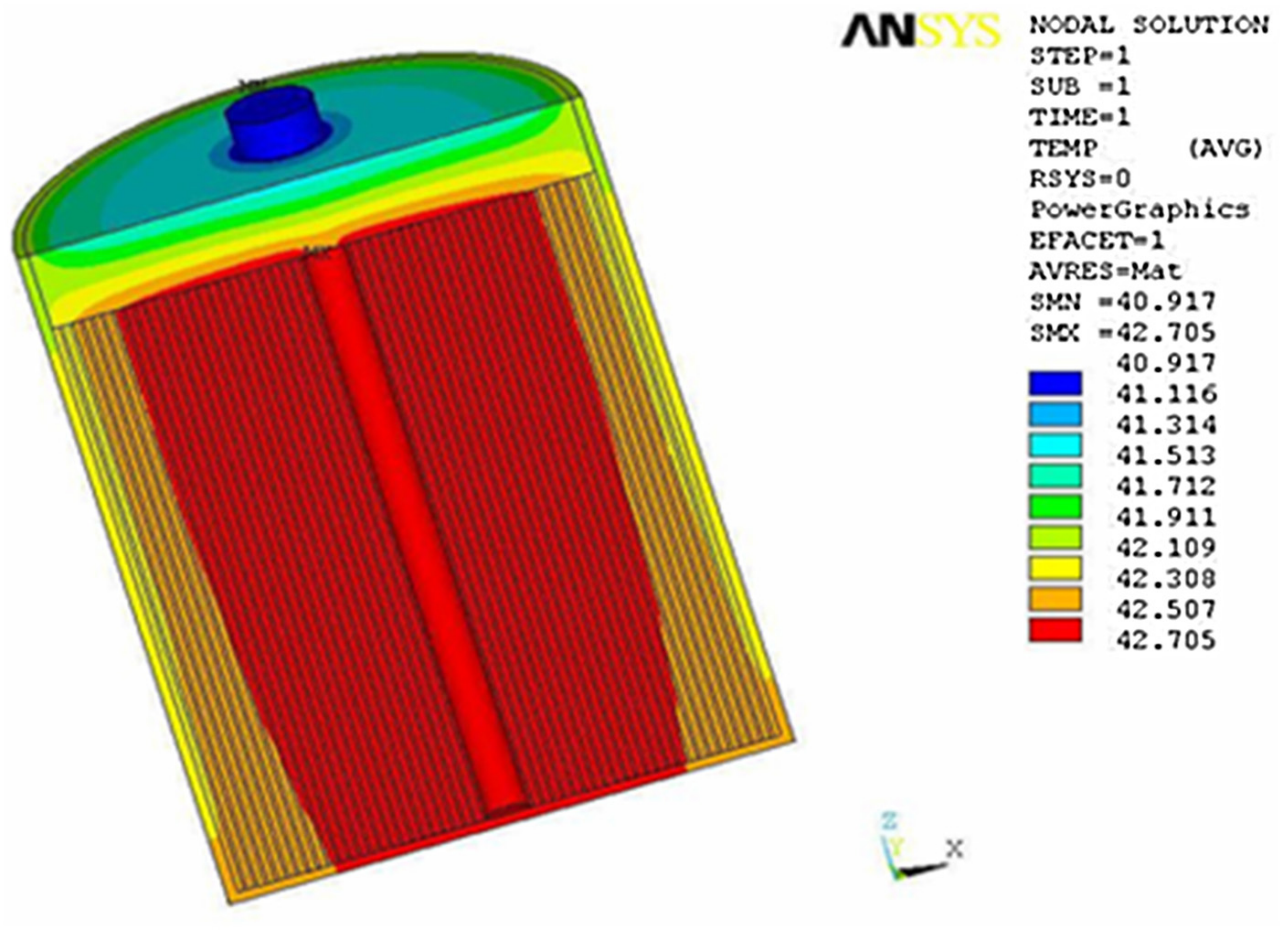
Temperature distribution at steady state.

Fig 5 is the temperature distribution of model supercapacitor after 5 cycles. Though heat is uniformly generated in core, the maximum temperature, 34.499°C, still appears there. It’s due to the reason that the innermost vacuum surface is approximately adiabatic. However, there are great heat convection and radiation between the outside surface of aluminum shell and surroundings, resulting in excellent heat dissipation. Compared with the temperature in core, the temperature of shell and adjacent region has decreased significantly. Fig 6 is the temperature distribution at steady state. The temperature distribution after 35 cycles is quite similar to that after 5 cycles, except that the temperature after 35 cycles is obviously enhanced. Furthermore, the maximum temperature still appears in core, reaching 42.7°C. Compared with the room temperature, the total working temperature rise of supercapacitor is about 17°C.

**Fig 7:**
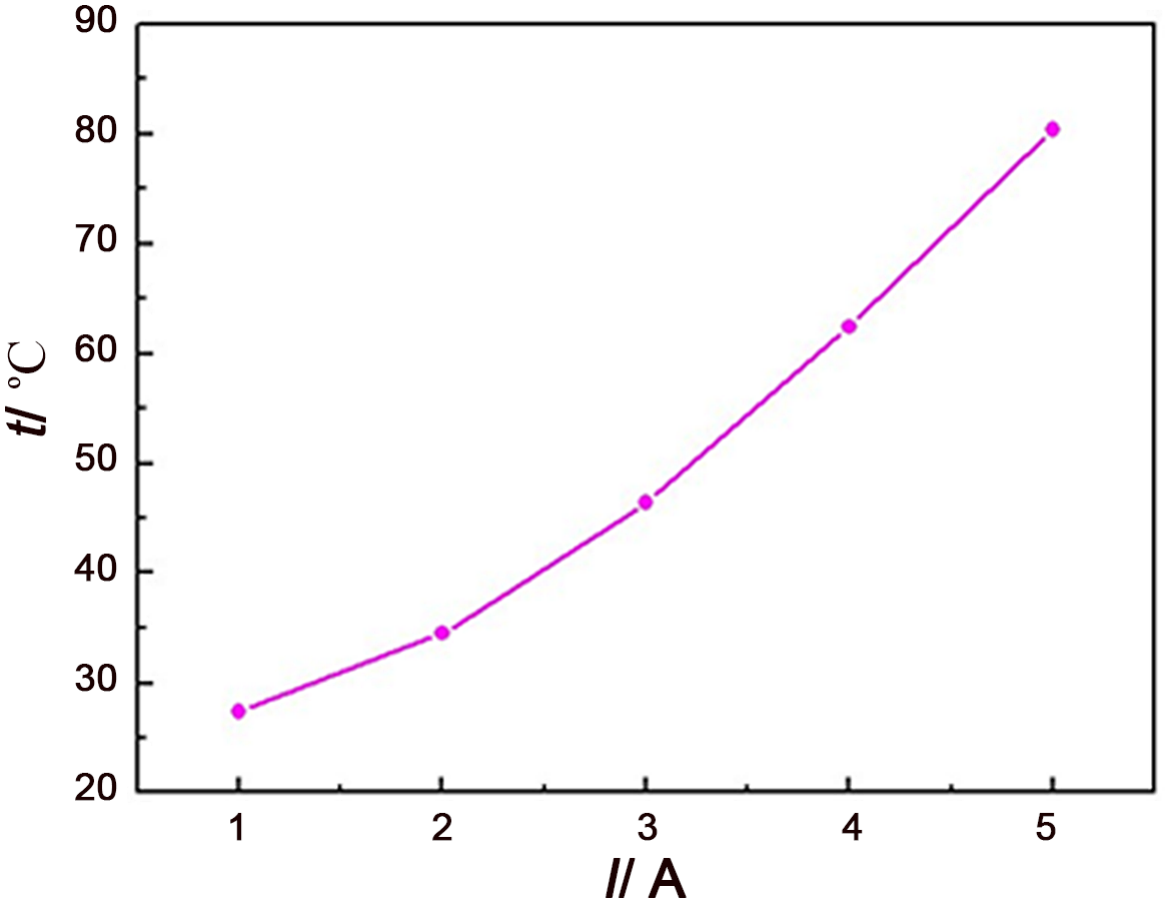
Changes of the maximal temperature in core area with the value of current.

To further study the relationship between the maximum temperature in core and charge-discharge current, 5 different currents from 1A to 5A have been adopted in experiments and the result is shown in Fig 7 (see S1 File for details). It shows that the temperature in core would rise rapidly with current increasing. If currents are 4A and 5A, the maximum temperatures can exceed 60°C and 80°C, respectively. Therefore, cooling measurements must be taken to make supercapacitor in top working state.

## Conclusions

Finite element analysis is adopted to analyze the distribution of inner temperature field of spiral wound supercapacitor in constant current charge-discharge experiment. Because heat is generated uniformly in core and the innermost vacuum surface is approximately adiabatic, the maximum temperature appears near the center in working process. We reach the conclusion that during 50 cycles at the constant current of 2A, the maximum temperature of inner area rises to 34.5°C after 5 cycles, and the final maximum temperature exceeds 42.5°C after 35 cycles. However, the temperature has not changed obviously in the following time, indicating that it’s in steady state. With working current increasing, the temperature of inner area would rise rapidly. Cooling measurements must be taken if the maximum temperature exceeds 60°C at the constant current of 4A. In the future, the thermal behavior analysis work is from the single supercapacitor extend to the group supercapacitor, and finally we write the relevant application software, which is used to predict and guide the application of supercapacitor.

## Supporting Information

S1 File S1 File is the Maximum temperature in core area with the change of cycle times. (DOC)

## Abbreviations

∇: is Laplace operator
*ρ*: is density
*C*_P_: is specific heat capacity
*λ*: is thermal conductivity
*P*: is local volume density
*θ*: is angular coordinate
*r*: is radial coordinate
*z*: is axial coordinate
*T*: is surface temperature
*T*_∞_: is ambient air temperature
*h*_conv_: is the convective heat transfer coefficient
*q*_conv_: is the heat flow rate per unit area of the convection heat transfer surface
