# Supplementary material for: Thermal Modelling Analysis of Spiral Wound Supercapacitor under Constant-Current Cycling
Source: PLoS One. 2015 Oct 7;10(10):e0138672. doi: 10.1371/journal.pone.0138672 (PMC4596575; doi:10.1371/journal.pone.0138672)
Supplement: S1 File — (DOC) [file pone.0138672.s001.doc]

Supporting Information

1. The geometric data

R1=0.001

R2=0.00118

R3=0.00148

R4=0.00166

R5=0.00196

R6=0.00214

R7=0.00244

R8=0.00262

R9=0.00292

R10=0.0031

R11=0.0034

R12=0.00358

R13=0.00388

R14=0.00406

R15=0.00436

R16=0.00454

R17=0.00484

R18=0.00502

R19=0.00532

R20=0.0055

R21=0.0058

R22=0.00598

R23=0.00628

R24=0.00646

R25=0.00676

R26=0.00694

R27=0.00724

R28=0.00742

R29=0.00772

R30=0.0079

R31=0.0082

R32=0.00838

R33=0.00868

R34=0.00886

R35=0.00916

R36=0.00934

R37=0.00964

R38=0.00982

R39=0.01012

R40=0.0103

R41=0.0106

R42=0.01078

R43=0.01108

R44=0.01126

R45=0.01156

R46=0.01174

R47=0.01204

R48=0.01222

R49=0.01252

L=0.031

2. Maximum temperature in core area with the change of cycle times

T=50s

S TS14

0.0000 25.774

0.22080E-03 25.775

0.44160E-03 25.776

0.66240E-03 25.776

0.88320E-03 25.776

0.11040E-02 25.777

0.13248E-02 25.777

0.15456E-02 25.777

0.17664E-02 25.777

0.19872E-02 25.777

0.22080E-02 25.777

0.24288E-02 25.777

0.26496E-02 25.777

0.28704E-02 25.776

0.30912E-02 25.776

0.33120E-02 25.776

0.35328E-02 25.776

0.37536E-02 25.775

0.39744E-02 25.775

0.41952E-02 25.775

0.44160E-02 25.774

0.46368E-02 25.774

0.48576E-02 25.773

0.50784E-02 25.773

0.52992E-02 25.772

0.55200E-02 25.771

0.57408E-02 25.771

0.59616E-02 25.770

0.61824E-02 25.769

0.64032E-02 25.769

0.66240E-02 25.768

0.68448E-02 25.767

0.70656E-02 25.766

0.72864E-02 25.765

0.75072E-02 25.764

0.77280E-02 25.763

0.79488E-02 25.762

0.81696E-02 25.760

0.83904E-02 25.759

0.86112E-02 25.757

0.88320E-02 25.756

0.90528E-02 25.754

***** PATH VARIABLE SUMMARY *****

S TS14

0.92736E-02 25.752

0.94944E-02 25.750

0.97152E-02 25.747

0.99360E-02 25.744

0.10157E-01 25.740

0.10378E-01 25.738

0.10598E-01 25.730

0.10819E-01 25.727

0.11040E-01 25.718

T=600s

PRINT ALONG PATH DEFINED BY LPATH COMMAND. DSYS= 0

***** PATH VARIABLE SUMMARY *****

S TS12

0.0000 32.491

0.22080E-03 32.491

0.44160E-03 32.492

0.66240E-03 32.492

0.88320E-03 32.492

0.11040E-02 32.492

0.13248E-02 32.492

0.15456E-02 32.492

0.17664E-02 32.492

0.19872E-02 32.491

0.22080E-02 32.490

0.24288E-02 32.489

0.26496E-02 32.489

0.28704E-02 32.487

0.30912E-02 32.487

0.33120E-02 32.485

0.35328E-02 32.484

0.37536E-02 32.483

0.39744E-02 32.482

0.41952E-02 32.480

0.44160E-02 32.478

0.46368E-02 32.477

0.48576E-02 32.475

0.50784E-02 32.474

0.52992E-02 32.471

0.55200E-02 32.470

0.57408E-02 32.467

0.59616E-02 32.465

0.61824E-02 32.462

0.64032E-02 32.460

0.66240E-02 32.457

0.68448E-02 32.454

0.70656E-02 32.452

0.72864E-02 32.448

0.75072E-02 32.446

0.77280E-02 32.441

0.79488E-02 32.439

0.81696E-02 32.433

0.83904E-02 32.431

0.86112E-02 32.425

0.88320E-02 32.421

0.90528E-02 32.415

***** PATH VARIABLE SUMMARY *****

S TS12

0.92736E-02 32.409

0.94944E-02 32.403

0.97152E-02 32.395

0.99360E-02 32.388

0.10157E-01 32.376

0.10378E-01 32.370

0.10598E-01 32.352

0.10819E-01 32.345

0.11040E-01 32.322

T=1200s

PRINT ALONG PATH DEFINED BY LPATH COMMAND. DSYS= 0

***** PATH VARIABLE SUMMARY *****

S TS10

0.0000 37.789

0.22080E-03 37.789

0.44160E-03 37.789

0.66240E-03 37.789

0.88320E-03 37.789

0.11040E-02 37.789

0.13248E-02 37.789

0.15456E-02 37.788

0.17664E-02 37.787

0.19872E-02 37.786

0.22080E-02 37.785

0.24288E-02 37.783

0.26496E-02 37.783

0.28704E-02 37.780

0.30912E-02 37.779

0.33120E-02 37.777

0.35328E-02 37.775

0.37536E-02 37.773

0.39744E-02 37.771

0.41952E-02 37.769

0.44160E-02 37.766

0.46368E-02 37.764

0.48576E-02 37.760

0.50784E-02 37.759

0.52992E-02 37.754

0.55200E-02 37.752

0.57408E-02 37.748

0.59616E-02 37.745

0.61824E-02 37.741

0.64032E-02 37.738

0.66240E-02 37.734

0.68448E-02 37.729

0.70656E-02 37.725

0.72864E-02 37.720

0.75072E-02 37.717

0.77280E-02 37.709

0.79488E-02 37.706

0.81696E-02 37.697

0.83904E-02 37.693

0.86112E-02 37.684

0.88320E-02 37.679

0.90528E-02 37.669

***** PATH VARIABLE SUMMARY *****

S TS10

0.92736E-02 37.661

0.94944E-02 37.652

0.97152E-02 37.640

0.99360E-02 37.631

0.10157E-01 37.613

0.10378E-01 37.605

0.10598E-01 37.578

0.10819E-01 37.567

0.11040E-01 37.534

T=1800s

PRINT ALONG PATH DEFINED BY LPATH COMMAND. DSYS= 0

***** PATH VARIABLE SUMMARY *****

S TS8

0.0000 41.579

0.22080E-03 41.579

0.44160E-03 41.579

0.66240E-03 41.579

0.88320E-03 41.579

0.11040E-02 41.578

0.13248E-02 41.578

0.15456E-02 41.577

0.17664E-02 41.576

0.19872E-02 41.574

0.22080E-02 41.573

0.24288E-02 41.571

0.26496E-02 41.570

0.28704E-02 41.567

0.30912E-02 41.566

0.33120E-02 41.563

0.35328E-02 41.561

0.37536E-02 41.558

0.39744E-02 41.555

0.41952E-02 41.553

0.44160E-02 41.549

0.46368E-02 41.547

0.48576E-02 41.542

0.50784E-02 41.540

0.52992E-02 41.534

0.55200E-02 41.532

0.57408E-02 41.526

0.59616E-02 41.523

0.61824E-02 41.517

0.64032E-02 41.513

0.66240E-02 41.508

0.68448E-02 41.503

0.70656E-02 41.498

0.72864E-02 41.491

0.75072E-02 41.487

0.77280E-02 41.478

0.79488E-02 41.474

0.81696E-02 41.463

0.83904E-02 41.458

0.86112E-02 41.447

0.88320E-02 41.440

0.90528E-02 41.428

***** PATH VARIABLE SUMMARY *****

S TS8

0.92736E-02 41.419

0.94944E-02 41.407

0.97152E-02 41.392

0.99360E-02 41.381

0.10157E-01 41.359

0.10378E-01 41.349

0.10598E-01 41.317

0.10819E-01 41.304

0.11040E-01 41.263

T=3600s

PRINT ALONG PATH DEFINED BY LPATH COMMAND. DSYS= 0

***** PATH VARIABLE SUMMARY *****

S TS17

0.0000 47.619

0.22080E-03 47.619

0.44160E-03 47.618

0.66240E-03 47.618

0.88320E-03 47.617

0.11040E-02 47.617

0.13248E-02 47.615

0.15456E-02 47.614

0.17664E-02 47.613

0.19872E-02 47.610

0.22080E-02 47.609

0.24288E-02 47.606

0.26496E-02 47.604

0.28704E-02 47.601

0.30912E-02 47.599

0.33120E-02 47.595

0.35328E-02 47.592

0.37536E-02 47.588

0.39744E-02 47.585

0.41952E-02 47.581

0.44160E-02 47.576

0.46368E-02 47.573

0.48576E-02 47.567

0.50784E-02 47.564

0.52992E-02 47.557

0.55200E-02 47.554

0.57408E-02 47.546

0.59616E-02 47.542

0.61824E-02 47.535

0.64032E-02 47.529

0.66240E-02 47.523

0.68448E-02 47.515

0.70656E-02 47.510

0.72864E-02 47.500

0.75072E-02 47.495

0.77280E-02 47.482

0.79488E-02 47.477

0.81696E-02 47.463

0.83904E-02 47.457

0.86112E-02 47.442

0.88320E-02 47.433

0.90528E-02 47.418

***** PATH VARIABLE SUMMARY *****

S TS17

0.92736E-02 47.405

0.94944E-02 47.390

0.97152E-02 47.371

0.99360E-02 47.357

0.10157E-01 47.329

0.10378E-01 47.316

0.10598E-01 47.275

0.10819E-01 47.257

0.11040E-01 47.204

T is at the steady mode.

PRINT ALONG PATH DEFINED BY LPATH COMMAND. DSYS= 0

***** PATH VARIABLE SUMMARY *****

S TS19

0.0000 51.108

0.22080E-03 51.107

0.44160E-03 51.107

0.66240E-03 51.107

0.88320E-03 51.106

0.11040E-02 51.105

0.13248E-02 51.103

0.15456E-02 51.102

0.17664E-02 51.100

0.19872E-02 51.097

0.22080E-02 51.096

0.24288E-02 51.092

0.26496E-02 51.091

0.28704E-02 51.086

0.30912E-02 51.084

0.33120E-02 51.080

0.35328E-02 51.077

0.37536E-02 51.072

0.39744E-02 51.068

0.41952E-02 51.064

0.44160E-02 51.058

0.46368E-02 51.055

0.48576E-02 51.048

0.50784E-02 51.045

0.52992E-02 51.036

0.55200E-02 51.033

0.57408E-02 51.024

0.59616E-02 51.019

0.61824E-02 51.011

0.64032E-02 51.005

0.66240E-02 50.997

0.68448E-02 50.989

0.70656E-02 50.982

0.72864E-02 50.971

0.75072E-02 50.966

0.77280E-02 50.952

0.79488E-02 50.946

0.81696E-02 50.929

0.83904E-02 50.923

0.86112E-02 50.905

0.88320E-02 50.896

0.90528E-02 50.879

***** PATH VARIABLE SUMMARY *****

S TS19

0.92736E-02 50.864

0.94944E-02 50.847

0.97152E-02 50.826

0.99360E-02 50.809

0.10157E-01 50.778

0.10378E-01 50.763

0.10598E-01 50.716

0.10819E-01 50.697

0.11040E-01 50.637
